# On Modern Progress in Ophthalmic Medicine and Surgery

**Published:** 1893-12-23

**Authors:** Robert Brudenell Carter

**Affiliations:** Consulting Ophthalmic Surgeon to St. George's Hospital


### On Modern Progress in Ophthalmic Medicine and Surgery.

By Robert Brudenell Carter, F.R.C.S., Consulting Ophthalmic Surgeon to St. George's Hospital.

Mr. Bowman's paper on "Iridectomy in Glaucoma," mentioned in my last, and read before the London meeting of the British Medical Association in 1862, was neither more nor less than an emphatic condemnation of the conduct of all those surgeons who, in suitable cases, failed to recommend and to perform the operation. The persons thus condemned were numerous and influential, and comprised not only Mr. Bowman's senior colleague, Mr. Dixon, but also a majority of the English ophthalmic specialists who were connected with hospitals other than the Royal London. For a considerable time, however, none of them took up the challenge, or attempted any defence of their position; and it was only under the pressure of public opinion that they were at last compelled to bestir themselves. They put forward as their first champion, oddly enough, the late Mr. Syme, then conspicuous as a general surgeon of great ability and of highly combative instincts, but who had probably never performed an eye operation in his life. His pronouncement on the question was succeeded by many others from various persons, in all of which iridectomy and its advocates were treated *de haut en bas*, and the proceeding itself was declared to be useless and unjustifiable. The opponents had just this much ground for their denunciations, that they appear to have meant by "glaucoma" only the fully-developed stage of the disease, in which sight was already lost and the case incurable. Some of them seem to have attempted to perform iridectomy in this condition, when there was not only no posibility of any good result, but when the changes which had occurred in the eye were of such a nature that any surgical interference was likely to be resented, and to give rise to subsequent pain and trouble. Others boasted that they had never performed the operation at all. The ignorance of eye disease which then prevailed among general practitioners had permitted men of very mediocre skill and knowledge to obtain baseless reputations as specialists; and it sometimes happened that, in their clumsy hands, iridectomy had less than fair play even in the conditions in which it was calculated to be useful. Although Mr. Bowman's paper had been read in August and was published in October, the controversy which it provoked did not break out until the autumn of the following year, when, for a few months, it became acute. Mr. Bowman himself maintained his part in it with great tact, dignity, and moderation, admitting that he did not understand in what way iridectomy diminished tension, but appealing to facts, and offering to exhibit a large number of cases, in defence of his conviction of the great value of the proceeding. His antagonists, as a rule, shrunk from any investigation of his facts, rested their opposition on what they were pleased to call "first principles," and finally, when driven from every other position, had recourse to the imputation of unworthy motives. From this time their collapse and defeat were inevitable; and, although they were strenuously supported by the *British Medical Journal*, which poured forth, week after week, shrill pæans in praise of ignorance and inefficiency, they soon ceased to make any impression. The efficacy and value of iridectomy were fully proved, and those who practised it were left at liberty to establish, on a firm basis, the conditions of its performance and of its utility. The controversy was almost forgotten when Mr. Dixon, some years later, recalled it to memory by a solemn public recantation of the views hostile to the operation which he had originally held and expressed.

I have dwelt at some length on the character of this discussion because it had very great influence in convincing the leaders of ophthalmology in this country of the pressing necessity for better teaching of the subject in the English schools of medicine, and it was therefore instrumental in the production of a great reform. In 1863 the opponents of iridectomy were able to presume on the ignorance of the bulk of the readers of the medical journals; and it was felt that steps should at once be taken by which this ignorance might be removed. To these steps, and to what followed from them, it will be necessary to return hereafter; but, in the meanwhile, it is perhaps permissible to support my own description of the character of the iridectomy controversy by a citation showing in what light it was regarded abroad. Dr. Testelin, of Lille, a very eminent ophthalmic surgeon, who was accustomed to analyse English publications for the Belgian *Annales d'Oculistique*, addressed a letter on the subject to Dr. Warlomont, the Editor of the *Annales*, in words so clear and forcible, that I will leave them untranslated. He said: "L'iridectomie appliquée à la cure du glaucome est depuis quelque temps, dans le *British Medical Journal*, l'objet d'une polémique des plus ardentes; et nous pourrions dire des plus affligeantes pour l'honneur de l'ophthalmologie anglaise, sans le talent hors ligne déployé par M. Bowman, pour la défense d'une des plus importantes conquêtes de la chirurgie moderne. Il m'était d'abord venu à la pensée de résumer cette discussion pour les lecteurs des Annales; mais elle est démesurément longue et je crois qu'elle ne leur apprendrait rien d'utile. D'une part, nous avons déjà publié la traduction de la belle leçon faite par M. Bowman, dans une des réunions de la Medical Association: de l'autre, il est par trop clair que les adversaires de ce praticien éminent ont pour partie pris de déprécier l'iridectomie appliquée au glaucome, parce que la découverte en est due à l'illustre Graefe; ensuite, parce que M. Bowman, déjà si riche de son propre fonds, a conquis un nouveau lustre par la façon brillante dont il a su se faire, en Angleterre, le promoteur de cette découverte. Je crois, au reste, qu'il arrivera de tout ceci un résultat autre que celui que l'on cherchait. Il est difficile d'admettre que la façon claire, méthodique et pleine de courtoisie en même temps que de vigueur, avec laquelle discute M. Bowman, n'ajoute pas à sa réputation et ne fasse pas triompher en Angleterre la cause de l'iridectomie, qui ne compte pas d'adversaire sur le continent. Il est encore plus difficile de supposer que la discussion passionnée et malveillante de ses antagonistes, que l'ignorance absolue (feinte ou réelle) qu'ils déploient, au sujet des découvertes de l'oculistique moderne, ne contribuent pas à jeter sur la cause qu'ils défendent le plus profond discrédit." When language such as the foregoing could be used by an authority of admitted skill and competence, it was impossible that the better teaching of ophthalmology could be long delayed.

